# Fulton County Medical Society

**Published:** 1873-04

**Authors:** 


*Fulton County Medical Society.*

The officers of this Society, elected at its late semi-annual meeting, are: W. T. Goldsmith, M.D., President; W. G. Owen, M.D., First Vice-President; E. J. Palmer, M.D., Second Vice-President; H. B. Lee, Secretary and Treasurer.

This Society is in a healthy and prosperous condition, with a large roll of members, and will, we think, be heard from, by contributions and reports, in the future issues of the RECORD.

